# Increased levels of the oxidative stress biomarker 8-iso-prostaglandin F_2α_ in wastewater associated with tobacco use

**DOI:** 10.1038/srep39055

**Published:** 2016-12-15

**Authors:** Yeonsuk Ryu, Emma Gracia-Lor, Richard Bade, J. A. Baz-Lomba, Jørgen G. Bramness, Sara Castiglioni, Erika Castrignanò, Ana Causanilles, Adrian Covaci, Pim de Voogt, Felix Hernandez, Barbara Kasprzyk-Hordern, Juliet Kinyua, Ann-Kathrin McCall, Christoph Ort, Benedek G. Plósz, Pedram Ramin, Nikolaos I. Rousis, Malcolm J. Reid, Kevin V. Thomas

**Affiliations:** 1 Norwegian Institute for Water Research (NIVA), Gaustadalléen 21, NO-0349 Oslo, Norway; 2 Norwegian Centre for Addiction Research, Faculty of Medicine, University of Oslo, PO box 1078 Blindern, 0316 Oslo, Norway; 3 IRCCS–Istituto di Ricerche Farmacologiche “Mario Negri”, Department of Environmental Health Sciences, Via La Masa 19, 20156, Milan, Italy; 4 Research Institute for Pesticides and Water, University Jaume I, Avda. Sos Baynat, E-12071 Castellón, Spain; 5 Department of Chemistry, University of Bath, Claverton Down, Bath BA2 7AY, UK; 6 KWR Watercycle Research Institute, Chemical Water Quality and Health, P.O. Box 1072, 3430 BB Nieuwegein, The Netherlands; 7 Toxicological Center, Department of Pharmaceutical Sciences, Campus Drie Eiken, University of Antwerp, Universiteitsplein 1, 2610 Wilrijk, Belgium; 8 Institute for Biodiversity and Ecosystem Dynamics (IBED), University of Amsterdam, Science Park 904, 1098 XH Amsterdam, The Netherlands; 9 Eawag, Swiss Federal Institute of Aquatic Science and Technology, CH 8600 Dübendorf, Switzerland; 10 Department of Environmental Engineering, Technical University of Denmark, Miljøvej, Building 115, 2800 Kgs. Lyngby, Denmark

## Abstract

Wastewater analysis has been demonstrated to be a complementary approach for assessing the overall patterns of drug use by a population while the full potential of wastewater-based epidemiology has yet to be explored. F_2_-isoprostanes are a prototype wastewater biomarker to study the cumulative oxidative stress at a community level. In this work, 8-iso-prostaglandin F_2α_ (8-iso-PGF_2α_) was analysed in raw 24 h-composite wastewater samples collected from 4 Norwegian and 7 other European cities in 2014 and 2015. Using the same samples, biomarkers of alcohol (ethyl sulfate) and tobacco (trans-3′-hydroxycotinine) use were also analysed to investigate any possible correlation between 8-iso-PGF_2α_ and the consumption of the two drugs. The estimated per capita daily loads of 8-iso-PGF_2α_ in the 11 cities ranged between 2.5 and 9.9 mg/day/1000 inhabitants with a population-weighted mean of 4.8 mg/day/1000 inhabitants. There were no temporal trends observed in the levels of 8-iso-PGF_2α_, however, spatial differences were found at the inter-city level correlating to the degree of urbanisation. The 8-iso-PGF_2α_ mass load was found to be strongly associated with that of trans-3′-hydroxycotinine while it showed no correlation with ethyl sulfate. The present study shows the potential for 8-iso-PGF_2α_ as a wastewater biomarker for the assessment of community public health.

Wastewater contains numerous endogenous and exogenous compounds excreted by humans as the result of metabolism. Some of these compounds can provide both direct and indirect information on specific diseases as well as the general health status of an individual^1^,^2^. The analysis of these biomarkers in urine has subsequently been established as diagnostic and prognostic tools for clinical research. Since in many developed countries the general population is connected to a sewer network, combining the urine from a defined population, this in theory facilitates the potential for analysing these biomarkers in wastewater as a means of providing information on the general health status of the said population^3^. An emerging field, wastewater-based epidemiology (WBE), relies on this concept and utilises wastewater as pooled urine as a source of information on public health. The usefulness of WBE has been demonstrated in the field of drug epidemiology by assessing the spatial and temporal trends of drug use in different communities^4^,^5^,^6^,^7^,^8^. Furthermore, there have been attempts to extend this approach for endogenously produced compounds that are directly associated with health and disease (e.g. oxidative stress^9^ and cancer^10^,^11^).

Oxidative stress is generally defined as the disruption in redox signalling and control, often caused by the imbalance in the level of prooxidants relative to antioxidants^12^. Oxidative stress is suspected to play a key role in the pathogenesis of various diseases including the top major causes of death in the world (ischemic heart disease^13^, stroke^14^, lower respiratory infections^15^,^16^ and chronic obstructive lung disease^17^). Consequently, a number of studies have focused on the measurement of oxidative stress, many of which are through specific biomarkers that indicate the oxidative damage^18^. Biomolecules such as lipid, protein and DNA are the targets of reactive oxygen/nitrogen species (generated by prooxidants) that are subsequently transformed into the unique compounds reflecting oxidative stress in the corresponding molecules (e.g. isoprostanes^19^, 3-nitrotyrosine^20^ and 8-hydroxy-2′-deoxyguanosine^21^).

Isoprostanes have been accepted as a reliable biomarker of oxidative stress and their use in clinical research is well established^22^. As isoprostanes originate from lipid membranes in every tissue of the human body, their levels provide a systematic and integrated measure of oxidative stress. Among the three major classes (F_2_-, D_2_- and E_2_-) of isoprostanes, F_2_-isoprostanes have been recognised as the most suitable biomarker since D_2_- and E_2_-isoprostanes are less stable^23^. F_2_-isoprostanes have been measured in a wide range of biological samples such as urine, plasma and exhaled breath condensate^24^. Furthermore, the potential of F_2_-isoprostanes as health biomarkers in WBE has been thoroughly reviewed based on biomarker selection criteria^25^. For a urinary biomarker to be suitable for use in WBE it needs to be stable in wastewater, excreted via urine at sufficiently high concentrations for quantification, specific to humans, and sensitive to the changes in the endpoint under investigation^25^. Subsequently, any compounds that meet these criteria can be used. Following an extensive literature review, F_2_-isoprostanes have been suggested as a prototype wastewater biomarker for public health assessment^25^ and thereafter, an analytical method for the most extensively studied F_2_-isoprostane isomer, 8-iso-prostaglandin F_2α_ (8-iso-PGF_2α_), in wastewater has been developed using immunoaffinity clean-up with liquid chromatography-high resolution mass spectrometry (IAC-LC-HRMS)^9^. In the latter study, 8-iso-PGF_2α_ was demonstrated to be stable in wastewater at least for 24 hours.

Association of oxidative stress with specific disease or health has been investigated in a number of studies that have reported the levels of F_2_-isoprostanes in urine^26^,^27^. The factors that have been correlated with urinary F_2_-isoprostane levels include various diseases such as diabetes^28^,^29^, cardiovascular diseases^30^ and cancer^31^ as well as lifestyles variables (e.g. smoking and alcohol consumption)^25^. It has been shown that smoking and alcohol consumption are the factors that most strongly correlate with increases of several folds in relative F_2_-isoprostane levels compared to controls^25^.

In WBE studies, tobacco use can be estimated by the analysis of two urinary metabolites of nicotine; cotinine and trans-3′-hydroxycotinine^32^,^33^,^34^,^35^,^36^. While enzymatic deconjugation is necessary to quantify total cotinine in wastewater, there has been no significant difference observed between measurements made with and without the enzyme treatment for trans-3′-hydroxycotinine^32^. When total concentrations are measured, both biomarkers provide very similar data for tobacco consumption^32^. The ethanol conjugates, ethyl glucuronide and ethyl sulfate are minor metabolites excreted into urine following alcohol intake^37^. Their suitability as wastewater biomarkers for estimation of alcohol consumption was first investigated by Reid and colleagues in 2011^38^. The study has demonstrated the high stability of ethyl sulfate in wastewater, in contrast to the rapid degradation of ethyl glucuronide^38^.

The aim of this work is to analyse 8-iso-PGF_2α_, in wastewater samples collected from 11 European cities and to investigate the possible correlation of 8-iso-PGF_2α_ levels to tobacco and alcohol uses. The temporal and spatial patterns of 8-iso-PGF_2α_ are evaluated to assess the suitability of this compound as a biomarker of population health in wastewater. The consumption of tobacco and alcohol has been measured in the same samples that were mentioned above using their specific biomarkers, trans-3′-hydroxycotinine and ethyl sulfate, respectively.

## Results

### 8-iso-PGF_2α_ analysis

**Figure 1:**
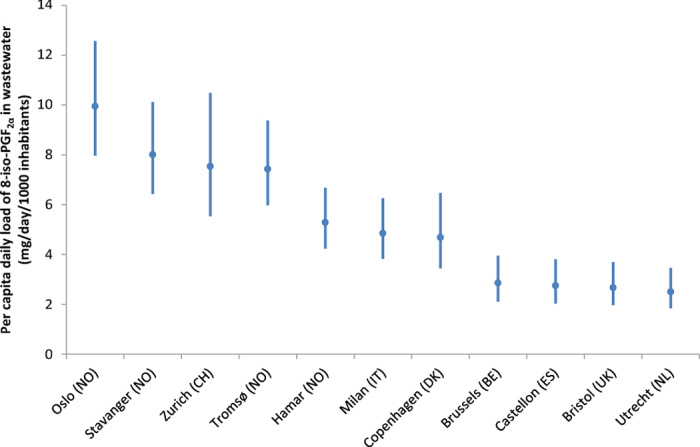
Estimated population-normalised daily loads of 8-iso-PGF_2α_ in the 11 cities. Estimates are presented as median with 95% credible interval based on the Monte Carlo simulations.

8-iso-PGF_2α_ was quantified in every wastewater sample with concentrations ranging from 8.7 to 18.0 ng/L (see Supplementary Table S1). The measured weekday and weekend loads in 11 cities (population-weighted mean) were 4.6 and 4.7 mg/day/1000 inhabitants, respectively. There was no significant difference (Wilcoxon, α = 0.05) between the weekday and weekend loads of 8-iso-PGF_2α_. Median per capita loads of 8-iso-PGF_2α_ in each city estimated by Monte Carlo simulation ranged from 2.5 to 9.9 mg/day/1000 inhabitants as shown in Fig. 1. Daily loads of 8-iso-PGF_2α_ in wastewater from the Norwegian cities (Oslo, Hamar, Stavanger and Tromsø) and Zurich were estimated to be the highest compared to all other cities. The estimated mean daily load from all the 11 cities (population-weighted) was 4.8 mg/day/1000 inhabitants (95% credible interval 4.3–5.4).

We also performed intra-city comparisons for Oslo using the previously reported levels of 8-iso-PGF_2α_ in the city^9^ in order to assess whether the load of this biomarker has changed over time. It was shown that there was no statistically significant difference between the loads measured in November 2014 (this study) and those in March 2015^9^ (Wilcoxon, α = 0.05). In addition, no weekly pattern (weekday vs. weekend) in the load could be observed in these intra-city comparisons (Wilcoxon, α = 0.05).

The total uncertainties, defined as the ratio between standard deviation and mean of a simulated value^7^,^39^, ranged between 12 and 17%. The overall uncertainty obtained was lower for the cities with longer periods of sampling (i.e. Norwegian cities and Milan) since there was little day-to-day variation in the loads of 8-iso-PGF_2α_ in each city, as well as the sampling error would be reduced by the square root of the number of samples^7^. In addition, the errors associated with population estimation and flow measurement were found to be the most dominant factors influencing the total uncertainty.

### Association with tobacco and alcohol consumption

**Figure 2:**
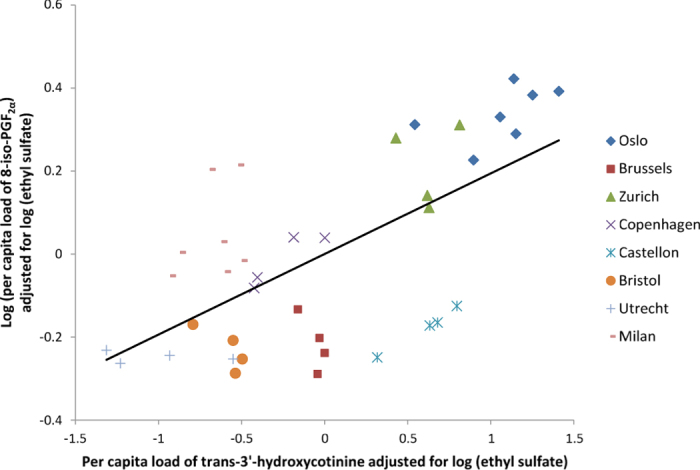
Partial regression plot between log 8-iso-PGF_2α_ and trans-3′-hydroxycotinine in the multiple linear regression model based on both weekday and weekend. 8-iso-PGF_2α_ data for Oslo was taken from our previous report^9^.

Multiple linear regression (MLR) analysis was performed to investigate the contribution of alcohol and tobacco consumption to oxidative stress level in the population. Table 1 presents the results of MLR analysis with log-transformed loads of 8-iso-PGF_2α_ as outcome and loads of trans-3′-hydroxycotinine together with log-transformed loads of ethyl sulfate as predictors. Even though the correlation was found to be stronger during weekdays, the MLR analysis consistently showed trans-3′-hydroxycotinine was statistically significant in all regression models, whereas log ethyl sulfate had no significant association with log 8-iso-PGF_2α_ (see Supplementary Fig. S1). As shown in Fig. 2, log 8-iso-PGF_2α_ is positively correlated with trans-3′-hydroxycotinine, with an increase of 19% for every increasing unit of trans-3′-hydroxycotinine load in wastewater.

The significant association observed between oxidative stress and tobacco use biomarkers in wastewater is in accordance with previous reports that have shown an increase in urinary 8-iso-PGF_2α_ concentrations induced by smoking. The average per capita loads of the two biomarkers calculated for each city were moderately correlated (ρ = 0.7, p = 0.07), which is also visible in Fig. 2 where the data points are generally clustered based on sampling location. Furthermore, an improved relationship was obtained when the observations from Milan were removed (ρ = 0.8, p = 0.02). The same trend (increase in correlation) was also observed in the MLR analysis for the data set excluding Milan (see Supplementary Fig. S2).

## Discussion

The present work is the first multi-city study reporting the measurement and comparison of the endogenous biomarker 8-iso-PGF_2α_ in wastewater. The measured values (8.7–18.0 ng/L) are in the range that is comparable with the concentrations reported in Oslo, March 2015 (18.9–23.3 ng/L)^9^. Even though a number of studies have measured the urinary level of 8-iso-PGF_2α_ in humans, most of them were either normalised against creatinine or only reported as concentrations that cannot be reliably compared with the values obtained in this study^25^. However, our estimates are in the similar range of the urinary 8-iso-PGF_2α_ levels reported as total daily mass (which can translate into 0.5–5 mg/day/1000 inhabitants)^25^ (Fig. 1).

The number of endogenous biomarkers excreted by humans that were successfully quantified in wastewater is very limited^9^,^40^,^41^. Furthermore, only one of those focused on an endogenous biomarker as an indicator for the overall health of population (8-iso-PGF_2α_)^9^ while another two studies investigated the use of endogenous biomarkers for estimating population size (5-hydroxyindoleacetic acid^40^ and DNA^41^). The development and validation of endogenous biomarkers for the assessment of public health through WBE is indeed complicated since there are a limited number of validated endogenous urinary biomarkers for routine clinical use. In comparison to exogenous biomarkers, produced following the exposure or consumption of specific substances, the selection of endogenous biomarkers is almost exclusively based on previous clinical studies. Therefore, F_2_-isoprostanes, selected following a thorough literature review^25^, are nonetheless the best candidate endogenous biomarker thus far, although it is certainly possible that there can be other suitable wastewater biomarkers to further support the feasibility of using WBE for monitoring community health. In addition, the extensive volume of relevant literature is another advantage of conducting further research on F_2_-isoprostanes for demonstrating the potential and amount of information that wastewater would possess in regards to community health.

As has been demonstrated in previous multi-city WBE studies, each community has different patterns of wastewater biomarkers related to lifestyle (e.g. drug use^6^,^7^). Our results are an extension of this concept and show that the level of a biomarker of health status, 8-iso-PGF_2α_, was distinguishable depending on the city with differences observed at both intra-and inter-national level. Furthermore, the level of 8-iso-PGF_2α_ was shown to be significantly correlated with trans-3′-hydroxycotinine, a biomarker of tobacco consumption, suggesting that the effect of smoking on oxidative stress can be observed at the population level through wastewater analysis. It should be also noted that a higher correlation between 8-iso-PGF_2α_ and trans-3′-hydroxycotinine was observed in MLR by excluding the results for Milan. Considering that 8-iso-PGF_2α_ in the samples from Milan was analysed in the country of origin, the importance of method comparability is highlighted in this instance^42^. These results nonetheless support the potential for 8-iso-PGF_2α_ to be used as a wastewater biomarker that can reflect the health status of the specific population connected by a sewer network.

The relationship between tobacco consumption and various negative health outcomes has been demonstrated in a number of studies. In particular, oxidative stress has been shown to be a central factor in the pathogenesis of the diseases related to tobacco consumption and lipid peroxidation is part of the mechanisms^43^,^44^. The lipid peroxidation biomarker, F_2_-isoprostanes, have been found to be present at levels several fold higher in the subjects exposed to both active and passive tobacco smoke^25^. The level of urinary 8-iso-PGF_2α_ is known to be sensitive to quitting and then restarting tobacco consumption^25^. At the population level, however, this change cannot be observed in a short time frame as the prevalence and the amount of tobacco consumption are rather consistent^33^,^34^. Accordingly, loads of trans-3′-hydroxycotinine as well as 8-iso-PGF_2α_ in each city from the present study were stable during the sampling period. On the contrary, alcohol consumption has a clear weekly pattern with an increase occurring during the weekend^45^. Even though the level of F_2_-isoprostanes in urine has been demonstrated to show a strong positive correlation with alcohol consumption including acute use^46^, the oxidative stress induced by alcohol consumption has not been visible in this study. This may be due to the fact that ethyl sulfate is excreted exclusively by the population that has consumed alcohol^47^ (mostly adults) while trans-3′-hydroxycotinine also reflects passive exposure to tobacco smoking^48^, thereby excreted by a larger population.

It was notable that a spatial difference for the 8-iso-PGF_2α_ per capita loads was observed within Norway in the same order as the size of the city’s population. Statistical tests revealed that Oslo has significantly higher 8-iso-PGF_2α_ level compared to the Norwegian cities of Tromsø and Hamar (Wilcoxon, α = 0.05) suggesting that the more urbanised region is likely to be exposed to higher oxidative stress. Unfortunately, the alcohol and tobacco biomarkers were not analysed in the other Norwegian samples than Oslo, and therefore it was not possible to see whether the 8-iso-PGF_2α_ loads adjusted for tobacco use would show the same trend. However, it is reasonable to hypothesise that the spatial variation is due to the degree of urbanisation as tobacco use is considered to be homogenous within Norway. Such an “urban factor” has been also reported in the previous studies carried out in Italy^49^,^50^.

Since F_2_-isoprostanes are a measure of collective oxidative stress as mentioned above, various other factors, not only tobacco consumption or urbanisation, can affect the urinary F_2_-isoprostanes. These variables include normal biological processes and therefore, our results should not be interpreted in a dichotomous way depending on the level of 8-iso-PGF_2α_ in wastewater sample (i.e. healthy vs. diseased community). Indeed, a longer period of monitoring is required to prove the added value of F_2_-isoprostanes as a wastewater biomarker of community health. The temporal and spatial trends measured over a substantially long time would then provide the reference intervals of 8-iso-PGF_2α_ in wastewater.

In conclusion, a reliable oxidative stress biomarker, 8-iso-PGF_2α_, has been quantified in the wastewater samples from 11 European cities. The estimated per capita load within city was relatively stable throughout the sampling period. Inter-city difference was observed, however the range of simulated values was comparable to the results from urinary analysis. A significant correlation of the oxidative stress biomarker with tobacco consumption was found by MLR analysis, as well as in Norway the level of 8-iso-PGF_2α_ increased depending on the degree of urbanisation. Our results support the hypothesis that wastewater is a pooled urine sample that reflects collective information for the health of the entire population. However, further studies are required to equivocally confirm the suitability of 8-iso-PGF_2α_ as a wastewater biomarker for community health assessment.

## Methods

### Wastewater samples

Wastewater was collected from 4 Norwegian and 7 other European cities in 2014 and 2015. Sample collection in Norway was conducted eight times per city (4 weekday + 4 weekend samples for each city) within approximately one month. Since weekend samples in the Norwegian cities were not available on daily basis, weekend sampling was performed for 72 h, from Friday to Sunday. Samples from the other European cities were collected four times within one week (2 weekday + 2 weekend samples) except for Milan where 7 consecutive daily samples were available. Samples were shipped frozen to Oslo and Milan for analysis. Details on the sampling can be found in the Supplementary Information (see Supplementary Table S1).

### Chemical analysis

The wastewater samples were analysed for 8-iso-PGF_2α_, trans-3′-hydroxycotinine and ethyl sulfate using previously validated methods^9^,^34^,^38^. All the methods include the use of deuterated internal standards and liquid chromatography coupled with mass spectrometry for ensuring appropriate quantification of target compounds in the complex matrix, wastewater. With the exception of the samples from Milan, 8-iso-PGF_2α_ analysis was conducted in Oslo by treating 100 mL of the sample with β-glucuronidase prior to IAC-LC-HRMS analysis^9^. Milan samples were prepared following the same protocol (β-glucuronidase treatment and IAC) but analysed by liquid chromatography coupled with tandem mass spectrometry (LC-MS/MS) in the country of origin. The details of LC-MS/MS analysis for 8-iso-PGF_2α_ in Milan can be found in Supplementary Table S2. The analysis of trans-3′-hydroxycotinine was carried out in Milan by extracting 3 mL of wastewater sample with HLB cartridge (3 mL, 60 mg) from Waters (Milford, MA, USA) before injecting into LC-MS/MS system^34^. Ethyl sulfate in wastewater samples was analysed in Oslo incorporating centrifugation (20,000 × *g*, 10 min) prior to ion-pair LC-MS/MS^38^. A summary of the validation data from the analytical methods employed in this study is reported in Supplementary Table S3.

### Back-calculations and data analysis

Daily mass loads of each biomarker were back-calculated by multiplying the concentrations in samples with the corresponding daily volumes of wastewater. Since the weekend samples from Norway were collected from Friday to Sunday as mentioned above, the weekend loads for Norwegian cities were divided by three to convert them into the average daily loads for weekend days. In order to compare the loads from cities with different population sizes, the daily mass loads were normalised by the wastewater catchment area’s population. Following previous studies^7^,^45^, Monte Carlo simulations (@RISK version 7.0.1, Palisade Corporation, Ithaca, NY, USA) were used to estimate daily mass loads of 8-iso-PGF_2α_. The overall uncertainty associated with the estimated per capita mass loads of 8-iso-PGF_2α_ was also assessed for each city by taking into account the errors of sampling, wastewater flow measurement, biomarker concentration, and population estimation. The systematic errors considered in the Monte Carlo simulation were as follows: 5% for sampling, 20% for flow measurement, precision of the analytical method (relative standard deviation) for the concentration and 20% for population size (see Supplementary Table S4 for details).

In addition, statistical analyses were carried out with SPSS Statistics 22.0 (SPSS Inc, Chicago, IL, USA) to compare average per capita loads of weekday with those of weekend and to assess the association of 8-iso-PGF_2α_ with drug biomarker level in wastewater. Log-transformation was executed prior to further analysis when the data was skewed.

## Additional Information

**How to cite this article**: Ryu, Y. *et al*. Increased levels of the oxidative stress biomarker 8-iso-prostaglandin F_2α_ in wastewater associated with tobacco use. *Sci. Rep.* **6**, 39055; doi: 10.1038/srep39055 (2016).

**Publisher's note:** Springer Nature remains neutral with regard to jurisdictional claims in published maps and institutional affiliations.

## Supplementary Material

Supplementary Information

## Figures and Tables

**Table 1:** Multiple linear regression results using log 8-iso-PGF_2α_ as dependent variable and log ethyl sulfate together with trans-3′-hydroxycotinine as independent variables.

Predictors	Total (n = 38)		Weekday (n = 19)		Weekend (n = 19)	
	Regression coefficient (95% confidence interval)	P value	Regression coefficient (95% confidence interval)	P value	Regression coefficient (95% confidence interval)	P value
Constant	0.32 (0.15, 0.48)	<10^−3^	0.29 (0.05, 0.54)	0.023	0.35 (0.08, 0.62)	0.015
Log (ethyl sulfate)	−0.13 (−0.35, 0.10)	0.268	−0.20 (−0.59, 0.20)	0.318	−0.08 (−0.44, 0.29)	0.654
Trans-3′-hydroxycotinine	0.19 (0.12, 0.27)	<10^−4^	0.22 (0.11, 0.34)	0.001	0.17 (0.04, 0.30)	0.016
	R^2^ = 0.42, adjusted R^2^ = 0.39, p < 10^−4^		R^2^ = 0.49, adjusted R^2^ = 0.44, p = 0.003		R^2^ = 0.36, adjusted R^2^ = 0.27, p = 0.037	
