# Supplementary material for: Increased levels of the oxidative stress biomarker 8-iso-prostaglandin F2α in wastewater associated with tobacco use
Source: Sci Rep. 2016 Dec 15;6:39055. doi: 10.1038/srep39055 (PMC5157025; doi:10.1038/srep39055)
Supplement: Supplementary Information [file srep39055-s1.pdf]

Supplementary Information

Increased levels of the oxidative stress biomarker 8-iso-prostaglandin $F_{2\alpha}$ in wastewater associated with tobacco use

Yeonsuk Ryu[1,2,*], Emma Gracia-Lor[3,4], Richard Bade[4], J.A. Baz-Lomba[1,2], Jørgen G. Bramness[2], Sara Castiglioni[3], Erika Castrignanò[5], Ana Causanilles[6], Adrian Covaci[7], Pim de Voogt[6,8], Felix Hernandez[4], Barbara Kasprzyk-Hordern[5], Juliet Kinyua[7], Ann-Kathrin McCall[9], Christoph Ort[9], Benedek G. Plósz[10], Pedram Ramin[10], Nikolaos I. Rousis[3], Malcolm J. Reid[1], and Kevin V. Thomas[1]

[1]Norwegian Institute for Water Research (NIVA), Gaustadalléen 21, NO-0349 Oslo, Norway

[2]Norwegian Centre for Addiction Research, Faculty of Medicine, University of Oslo, PO box 1078 Blindern, 0316 Oslo, Norway

[3]IRCCS–Istituto di Ricerche Farmacologiche "Mario Negri", Department of Environmental Health Sciences, Via La Masa 19, 20156, Milan, Italy

[4]Research Institute for Pesticides and Water, University Jaume I, Avda. Sos Baynat, E-12071 Castellón, Spain

[5]Department of Chemistry, University of Bath, Claverton Down, Bath BA2 7AY, UK

[6]KWR Watercycle Research Institute, Chemical Water Quality and Health, P.O. Box 1072, 3430 BB Nieuwegein, The Netherlands

[7]Toxicological Center, Department of Pharmaceutical Sciences, Campus Drie Eiken, University of Antwerp, Universiteitsplein 1, 2610 Wilrijk, Belgium

[8]Institute for Biodiversity and Ecosystem Dynamics (IBED), University of Amsterdam, Science Park 904, 1098 XH Amsterdam, The Netherlands

[9]Eawag, Swiss Federal Institute of Aquatic Science and Technology, CH 8600 Dübendorf, Switzerland

[10]Department of Environmental Engineering, Technical University of Denmark, Miljøvej, Building 115, 2800 Kgs. Lyngby, Denmark

[*]Corresponding author. E-mail: yeonsuk.ryu@niva.no, Tel: +47 22 18 51 00

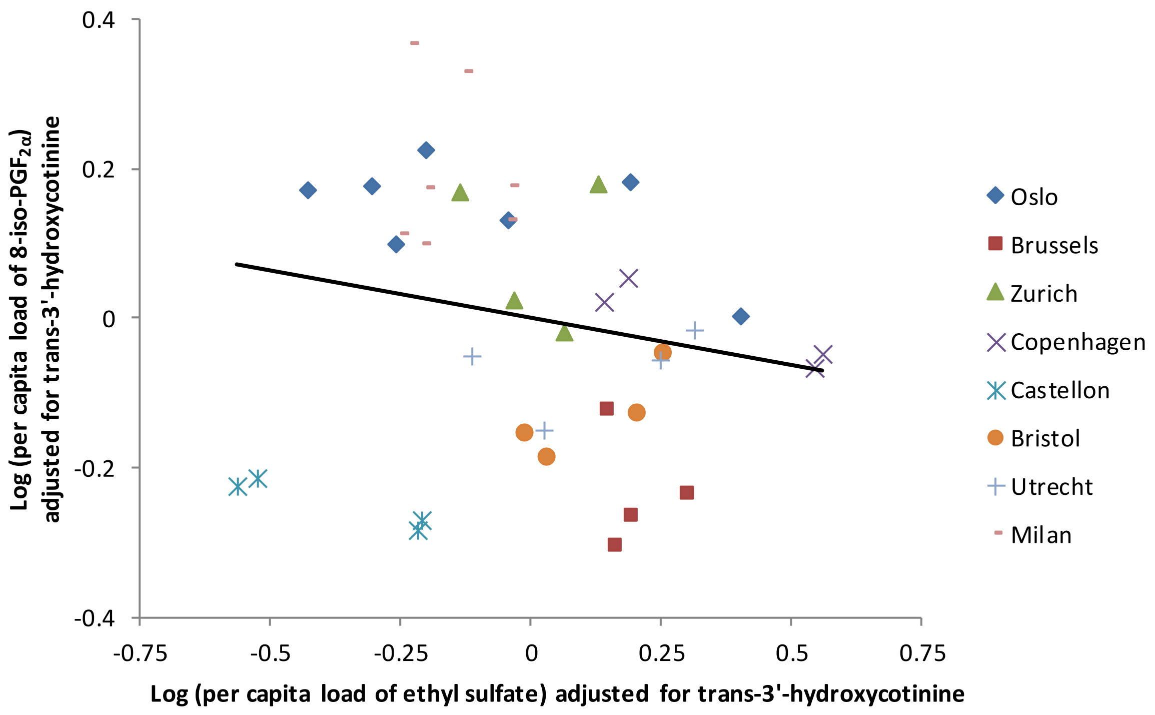

Supplementary Figure S1. Partial regression plot between log 8-iso-$PGF_{2\alpha}$ and log ethyl sulfate from the multiple linear regression analysis based on both weekday and weekend. 8-iso-$PGF_{2\alpha}$ data for Oslo was taken from our previous report[1].

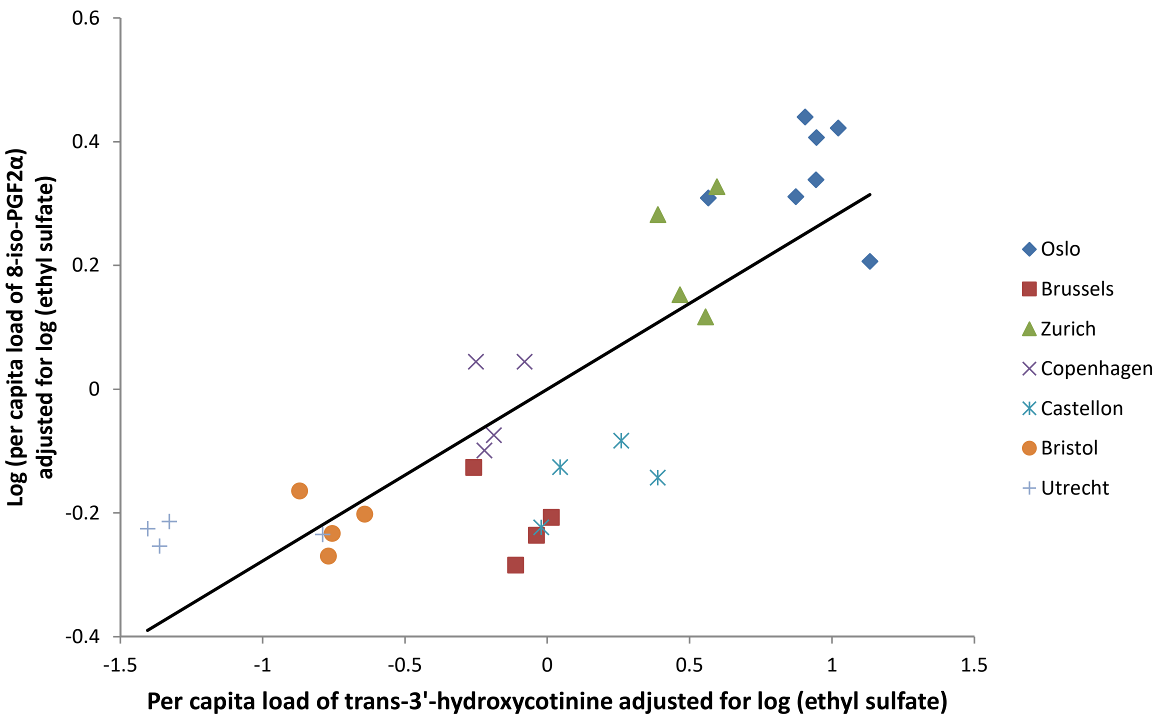

Supplementary Figure S2. Partial regression plot between log 8-iso-$PGF_{2\alpha}$ and trans-3'-hydroxycotinine from the multiple linear regression analysis performed after excluding Milan data. 8-iso-$PGF_{2\alpha}$ data for Oslo was taken from our previous report[1].

Supplementary Table S1. Concentration of biomarkers and sewage flow in participating cities during the sampling period.

City	Population	Sampling start	Sampling end	Day	Sewage volume (m^3/day)	8-iso-PGF$_{2\alpha}$ (ng/L)	ethyl sulfate (ng/mL)	trans-3′-hydroxycotinine (ng/mL)
Brussels (BE)	953987	20/03/2015	21/03/2015	Fri	234906	10.6	22.4	9.0
	953987	21/03/2015	22/03/2015	Sat	233096	11.9	29.8	9.5
	953987	23/03/2015	24/03/2015	Mon	234774	9.4	20.3	8.6
	953987	24/03/2015	25/03/2015	Tue	359951	8.7	12.0	5.2
Zurich (CH)	410000	18/03/2015	19/03/2015	Wed	157084	15.2	12.9	7.3
	410000	19/03/2015	20/03/2015	Thu	161005	15.4	9.6	6.8
	410000	21/03/2015	22/03/2015	Sat	200010	18.0	6.3	5.6
	410000	22/03/2015	23/03/2015	Sun	243013	14.6	9.3	4.5
Copenhagen (DK)	531000	10/03/2015	11/03/2015	Tue	150936	17.2	16.9	7.6
	531000	11/03/2015	12/03/2015	Wed	147175	17.8	18.2	7.2
	531000	14/03/2015	15/03/2015	Sat	137793	16.6	49.5	8.6
	531000	15/03/2015	16/03/2015	Sun	137244	15.6	47.5	8.4
Castellon (ES)	180690	27/03/2015	28/03/2015	Fri	43728	11.9	9.9	10.3
	180690	28/03/2015	29/03/2015	Sat	38301	13.8	5.0	10.4
	180690	30/03/2015	31/03/2015	Mon	37469	11.2	9.9	9.9
	180690	31/03/2015	01/04/2015	Tue	40476	11.5	4.0	8.8
Milan (IT)	1100000	04/02/2015	05/02/2015	Wed	403960	9.9	4.1[b]	2.7
	1100000	05/02/2015	06/02/2015	Thu	673970	11.0	3.1[b]	1.9
	1100000	06/02/2015	07/02/2015	Fri	660810	10.6	2.3[b]	1.4
	1100000	07/02/2015	08/02/2015	Sat	469900	9.8	5.0[b]	2.1
	1100000	08/02/2015	09/02/2015	Sun	395410	10.2	5.7[b]	2.3
	1100000	09/02/2015	10/02/2015	Mon	423340	11.2	3.9[b]	2.5
	1100000	10/02/2015	11/02/2015	Tue	424210	10.0	3.6[b]	2.7
Utrecht (NL)	300000	04/03/2015	05/03/2015	Wed	47740	14.6	10.7	4.5
	300000	05/03/2015	06/03/2015	Thu	45030	15.9	18.9	9.0

	300000	07/03/2015	08/03/2015	Sat	46030	15.7	26.4	5.5
	300000	08/03/2015	09/03/2015	Sun	46900	16.7	29.8	5.3
Hamar (NO)	55000	14/11/2014	17/11/2014	Fri-Sun	66207	10.3	NA[c]	NA[c]
	55000	17/11/2014	18/11/2014	Mon	24881	11.8	NA[c]	NA[c]
	55000	21/11/2014	24/11/2014	Fri-Sun	80516	9.6	NA[c]	NA[c]
	55000	25/11/2014	26/11/2014	Tue	42669	9.4	NA[c]	NA[c]
	55000	28/11/2014	01/12/2014	Fri-Sun	80488	10.5	NA[c]	NA[c]
	55000	03/12/2014	04/12/2014	Wed	25153	11.9	NA[c]	NA[c]
	55000	05/12/2014	08/12/2014	Fri-Sun	63992	11.3	NA[c]	NA[c]
	55000	11/12/2014	12/12/2014	Thu	21655	11.3	NA[c]	NA[c]
Oslo (NO)	580639	05/11/2014	06/11/2014	Wed	491702	14.8	NA[c]	NA[c]
	580639	07/11/2014	10/11/2014	Fri-Sun	1313833	14.6	NA[c]	NA[c]
	580639	12/11/2014	13/11/2014	Wed	416186	15.6	NA[c]	NA[c]
	580639	14/11/2014	17/11/2014	Fri-Sun	1299286	15.3	NA[c]	NA[c]
	580639	19/11/2014	20/11/2014	Wed	268451	15.1	NA[c]	NA[c]
	580639	21/11/2014	24/11/2014	Fri-Sun	851342	17.0	NA[c]	NA[c]
	580639	26/11/2014	27/11/2014	Wed	361952	14.6	NA[c]	NA[c]
	580639	28/11/2014	01/12/2014	Fri-Sun	779316	13.9	NA[c]	NA[c]
	580639	11/03/2015	12/03/2015	Wed	332093	19.8[a]	5.1[b]	5.3
	580639	12/03/2015	13/03/2015	Thu	306596	19.1[a]	4.3[b]	5.8
	580639	13/03/2015	14/03/2015	Fri	275737	20.0[a]	9.1[b]	6.6
	580639	14/03/2015	15/03/2015	Sat	255126	21.6[a]	15.4[b]	6.5
	580639	15/03/2015	16/03/2015	Sun	248717	19.4[a]	32.0[b]	8.3
	580639	16/03/2015	17/03/2015	Mon	252838	18.9[a]	5.7[b]	6.9
	580639	17/03/2015	18/03/2015	Tue	251013	23.3[a]	4.0[b]	7.1
Stavanger (NO)	240000	24/10/2014	27/10/2014	Fri-Sun	493562	13.9	NA[c]	NA[c]
	240000	06/11/2014	07/11/2014	Thu	118568	17.0	NA[c]	NA[c]
	240000	07/11/2014	10/11/2014	Fri-Sun	393481	15.5	NA[c]	NA[c]
	240000	17/11/2014	18/11/2014	Mon	87215	14.3	NA[c]	NA[c]
	240000	21/11/2014	24/11/2014	Fri-Sun	311918	16.8	NA[c]	NA[c]

	240000	27/11/2014	28/11/2014	Thu	118400	15.9	NA[c]	NA[c]
	240000	02/12/2014	03/12/2014	Tue	84802	17.7	NA[c]	NA[c]
	240000	05/12/2014	08/12/2014	Fri-Sun	397626	16.0	NA[c]	NA[c]
Tromsø (NO)	20000	04/02/2015	05/02/2015	Wed	6872	15.0	NA[c]	NA[c]
	20000	06/02/2015	09/02/2015	Fri-Sun	28507	16.4	NA[c]	NA[c]
	20000	10/02/2015	11/02/2015	Tue	11327	16.2	NA[c]	NA[c]
	20000	13/02/2015	16/02/2015	Fri-Sun	21276	17.4	NA[c]	NA[c]
	20000	18/02/2015	19/02/2015	Wed	8059	15.9	NA[c]	NA[c]
	20000	20/02/2015	23/02/2015	Fri-Sun	33915	15.0	NA[c]	NA[c]
	20000	24/02/2015	25/02/2015	Tue	7973	16.9	NA[c]	NA[c]
	20000	27/02/2015	02/03/2015	Fri-Sun	27837	16.0	NA[c]	NA[c]
Bristol (UK)	886650	12/03/2015	13/03/2015	Thu	197523	13.6	21.8	6.1
	886650	14/03/2015	15/03/2015	Sat	220687	11.1	18.6	6.4
	886650	15/03/2015	16/03/2015	Sun	193194	10.1	13.4	6.3
	886650	16/03/2015	17/03/2015	Mon	197493	10.6	11.9	6.1

[a]Data from Ryu et al. (2015)[1]
[b]Data from Ryu et al. (2016)[2]
[c]Not analysed.

Supplementary Table S2. LC-MS/MS conditions for the analysis of 8-iso-$PGF_{2\alpha}$ in Milan samples.

HPLC pump	Agilent 1200 Series (Santa Clara, CA, USA)		Mass Spectrometer	API 5500 QqQ (AB Sciex, Thornhill, Ontario, Canada)			
Autosampler	4 °C		Interface	Turbo Ion Spray			
Injection volume	4 µL		Polarity	Negative			
Column	XSELECT^{TM} CSH^{TM} C18 column (2.1 × 100 mm, 2.5 µm) (Waters, Milford, MA, USA)		Scan type	Multiple reaction monitoring (MRM)			
Column oven	Room temperature		Ion spray voltage (IS)	-4500 V			
Eluent A	0.05% acetic acid in water		Source temperature	350 °C			
Eluent B	acetonitrile		Curtain gas (CUR)	25			
Flow rate	0.16 mL/min		Collision gas (CAD)	7			
Gradient	Linear		Ion source gas 1 (GS 1)	40			
Composition	Time (min)	%B	Ion source gas 2 (GS 2)	45			
	0	2	Declustering potential (DP)	-80			
	5	70	Entrance potential (EP)	-10			
	9	98	Compound	Precursor ion	CXP[a]	CE[b]	Product ion
	13	98	8-iso-$PGF_{2\alpha}$	m/z 353	-13	-34	m/z 193
	14	2				-31	m/z 247
	21	2	8-iso-$PGF_{2\alpha}$-d4	m/z 357	-15	-35	m/z 197

[a]Collision cell exit potential.

[b]Collision energy.

Supplementary Table S3. Summary of validation results from the analytical methods used in the present study.

Compound	Linear range (ng/mL)	R^2	LOQ[a] (ng/L)	Recovery (%)	RSD[b] (%)	Reference
8-iso-PGF$_{2\alpha}$	0.1 – 100	0.999	0.3	103 – 113	3 – 7	Ryu et al. (2015)[1]
trans-3′-hydroxycotinine	0 – 600	0.999	1.9	87	8	Senta et al. (2015)[3]
ethyl sulfate	2 – 200	0.998	1000	98	7	Reid et al. (2011)[4]

[a]Limit of quantification
[b]Relative standard deviation

Supplementary Table S4. Parameters used in Monte Carlo simulations to estimate daily mass loads (mg/day/1000 inhabitants) of 8-iso-$PGF_{2\alpha}$[a].

	Sampling uncertainty (U_s)	Measured Concentration (C)	Daily volume of wastewater (V)	Population (P)	Per capita daily mass load (L)	Total uncertainty (U_t)
Norwegian cities	Normal (1, $\frac{0.05}{\sqrt{8}}$)	Normal (C, 0.068*C)				
Milan	Normal (1, $\frac{0.05}{\sqrt{7}}$)	Normal (C, 0.068*C)	Normal (V, 0.2*V)	Normal (P, 0.2*P)	$U_s \cdot \frac{C \cdot V}{P}$	$\frac{SD (L)}{Mean (L)}$
The rest	Normal (1, $\frac{0.05}{\sqrt{4}}$)	Normal (C, 0.068*C)				

[a]Normal (mean, SD) specifies a normal distribution with the entered mean and standard deviation.

References

1. Ryu, Y., Reid, M. J. & Thomas, K. V. Liquid chromatography–high resolution mass spectrometry with immunoaffinity clean-up for the determination of the oxidative stress biomarker 8-iso-prostaglandin F2alpha in wastewater. *J. Chromatogr. A* **1409,** 146–151 (2015).
2. Ryu, Y. *et al.* Comparative measurement and quantitative risk assessment of alcohol consumption through wastewater-based epidemiology: An international study in 20 cities. *Sci. Total Environ.* doi:10.1016/j.scitotenv.2016.04.138
3. Senta, I., Gracia-Lor, E., Borsotti, A., Zuccato, E. & Castiglioni, S. Wastewater analysis to monitor use of caffeine and nicotine and evaluation of their metabolites as biomarkers for population size assessment. *Water Res.* **74,** 23–33 (2015).
4. Reid, M. J., Langford, K. H., Mørland, J. & Thomas, K. V. Analysis and Interpretation of Specific Ethanol Metabolites, Ethyl Sulfate, and Ethyl Glucuronide in Sewage Effluent for the Quantitative Measurement of Regional Alcohol Consumption. *Alcohol. Clin. Exp. Res.* **35,** 1593–1599 (2011).
